# Determination of Benefits of Cochlear Implantation in Children with Auditory Neuropathy

**DOI:** 10.1371/journal.pone.0127566

**Published:** 2015-05-26

**Authors:** Fei Ji, Jianan Li, Mengdi Hong, Aiting Chen, Qingshan Jiao, Li Sun, Sichao Liang, Shiming Yang

**Affiliations:** Department of Otolaryngology-Head and Neck Surgery, Institute of Otolaryngology, Chinese People’s Liberation Army General Hospital, Beijing 100853, China; University of California, Irvine, UNITED STATES

## Abstract

**Background:**

Auditory neuropathy (AN) is a recently recognized hearing disorder characterized by intact outer hair cell function, disrupted auditory nerve synchronization and poor speech perception and recognition. Cochlear implants (CIs) are currently the most promising intervention for improving hearing and speech in individuals with AN. Although previous studies have shown optimistic results, there was large variability concerning benefits of CIs among individuals with AN. The data indicate that different criteria are needed to evaluate the benefit of CIs in these children compared to those with sensorineural hearing loss. We hypothesized that a hierarchic assessment would be more appropriate to evaluate the benefits of cochlear implantation in AN individuals.

**Methods:**

Eight prelingual children with AN who received unilateral CIs were included in this study. Hearing sensitivity and speech recognition were evaluated pre- and postoperatively within each subject. The efficacy of cochlear implantation was assessed using a stepwise hierarchic evaluation for achieving: (1) effective audibility, (2) improved speech recognition, (3) effective speech, and (4) effective communication.

**Results:**

The postoperative hearing and speech performance varied among the subjects. According to the hierarchic assessment, all eight subjects approached the primary level of effective audibility, with an average implanted hearing threshold of 43.8 ± 10.2 dB HL. Five subjects (62.5%) attained the level of improved speech recognition, one (12.5%) reached the level of effective speech, and none of the subjects (0.0%) achieved effective communication.

**Conclusion:**

CIs benefit prelingual children with AN to varying extents. A hierarchic evaluation provides a more suitable method to determine the benefits that AN individuals will likely receive from cochlear implantation.

## Introduction

Auditory neuropathy (AN) is a recently recognized hearing disorder primarily characterized by disrupted auditory nerve synchronization, intact outer hair cell function, and poor speech recognition [1–5]. AN may involve the inner hair cells, ribbon synapses, and the stem of cranial nerve VIII. The incidence of AN among hearing-impaired neonates is approximately 10% [6–8] or higher [9]. Great challenges exist in the treatment of AN. According to the currently available clinical evidence, medication and acoustic amplification have limited benefits [10, 11]. Cochlear implantation is now recognized as the most promising intervention for improving audibility and speech communication in individuals with AN. However, as the mechanism of AN is not well understood, there is debate concerning the benefits of cochlear implants (CIs) in children with this type of hearing loss.

Cochlear implantation used to be contraindicated for AN due to its association with nerve degeneration secondary to processes such as demyelination and axon impairment [1]. However, evidence from animal experiments has shown that CIs can restore synchronization through electrical stimulation [12, 13]. Miyamoto *et al*. [14] and Trautwein *et al*. [15] reported the earliest attempts of using CIs for AN, and demonstrated limited efficacy. Subsequent studies demonstrated similar postoperative performance after implantation between children with AN and those with sensorineural hearing loss (SNHL) [16–26]. Based on this evidence, cochlear implantation has been advocated as a feasible treatment for AN [11, 27]. However, some reports show poorer outcomes in AN subjects compared to SNHL recipients [22, 24, 28–31], and others demonstrate variable outcomes [11, 25, 30]. Although variability has also been observed in SNHL recipients, the variability in implanted prelingual AN children is thought to relate to uncertainty of the lesion location.

Various criteria have been used to evaluate the benefit of CIs in individuals with AN. Researchers have drawn both positive [15, 17–19] and negative [14, 28–30, 32, 33] conclusions based on the various postoperative results, including: (1) distinct neural responses of the peripheral auditory neural pathway evoked by the CI, including the electrically evoked auditory brain stem response (EABR), electrically evoked compound action potential (ECAP), and electrical stapedius reflex [15, 18, 19, 34, 35]; (2) improved hearing thresholds in the speech frequency range [19, 33]; and (3) improved speech recognition performance [22, 25, 36] with hearing aids [17], or to a level equivalent to that achieved by SNHL subjects s [16, 21, 23, 37, 38].

Speech recognition is the main functional impairment of AN, and a substantial challenge in clinical management of AN individual is hearing restoration. The most well-established and useful evaluation methods for identifying CI candidates assess the individual’s speech recognition capability. The results of evaluations from before and after implantation can be compared to determine the quantitative changes in speech recognition [22, 25, 36], and to determine the differences between subjects treated with CIs and hearing aids [17], or between subjects with AN and SNHL [16, 21, 23, 37, 38]. Because individuals with AN can exhibit different levels of language development and cooperation with subjective speech tests, various methods are available to evaluate speech performance, including open-set recognition tests and scales. The open-set speech recognition tests include the Consonant-nucleus-consonant Monosyllabic Words Test [22], Paden–Brown Phonologic Kit [20], Hearing in Noise Test [16], Melbourne Speech Perception Score [23, 24], Glendonald Auditory Screening Procedure [26], and the Common Phrases Test [21]. Scales include the Meaningful Auditory Integration Scale (MAIS), Infant-Toddler MAIS (IT-MAIS) [19, 25, 26], Meaningful Use of Speech Scale [17], and the Categories of Auditory Performance Scale [21]. Indeed, several studies have used these methods concurrently to comprehensively evaluate the efficacy of CIs in individuals with AN [19, 37].

Given the diversity in available evaluation methods, a proper criterion for determining the benefits of CIs in AN individuals is needed. This could be accomplished by using a stepwise hierarchic assessment that demonstrates the effects of cochlear implantation by rating categories or capability levels according to various psychophysic and physiologic results. This hierarchic assessment could be based on functional performance, including hearing sensitivity, speech recognition, language skills, communication skills, and even academic or social skills [11]. Various assessment methods could be integrated into such an open-framework. In the present study, a hierarchic assessment is proposed and evaluated for determining the benefits that AN children obtain from cochlear implantation.

## Materials and Methods

### Inclusion and exclusion criteria

Diagnostic test sets for AN, including pure tone audiometry, auditory brain stem response (ABR), cochlear microphonic (CM), distortion-product otoacoustic emissions (DPOAEs), tympanometry, and stapedial acoustic reflex, were performed before implantation to determine whether a subject was eligible for inclusion in the study. Visual reinforcement audiometry or play audiometry was used for children too young for the standard pure tone audiometry examination.

The following inclusion criteria were used in the present study: (1) prelingual children who underwent cochlear implantation at < 5 years of age; (2) normal tympanic membranes upon otoscopic examination; (3) presence of nonconductive hearing loss according to audiogram and tympanogram results; (4) absent or seriously abnormal ABR waveforms or ABR thresholds that are disproportionate to the pure tone behavior thresholds; (5) presence of DPOAEs or CM, and (6) presence of CM and type A tympanogram in subjects without DPOAEs.

The following exclusion criteria were used in the present study: (1) not meeting the aforementioned inclusion criteria; (2) having a CI that had been switched on for no more than 1 mo or a CI that had never been switched on before the data analysis; and (3) loss to follow-up.

### Subjects

In total, ten prelingual AN children received CIs in our study. However, two of these children (ANCI_06 and 08) were removed from the study according to the exclusion criteria, and their data were not included in the analysis. Thus, data from eight prelingual children (three female and five male) with AN who met the inclusion criteria were analyzed (Table 1). All subjects received unilateral CIs at a mean age of 30.25 ± 13.60 months (Table 2). Preoperative magnetic resonance imaging showed a normal cochlear nerve in all subjects with the exception of subject ANCI_02. Behavior audiometry showed bilateral severe hearing loss in one subject (ANCI_10) and profound hearing loss in five subjects (ANCI_01, 03, 04, 05, and 09). The remaining two children (ANCI_02 and 07) could not cooperate with the behavior audiometry examination before implantation (Table 3). Two children (ANCI_04 and 09) used hearing aids both before and after the implantation, and three children (ANCI_04, 05, and 07) had attended normal kindergarten at the time of data collection.

**Table 1:** Subjects’ clinical diagnostic results.

Subject	Sex	Ear	ABR	DPOAEs	CM	Tympanogram	Reflex	MRI
ANCI_01	Male	L	Absent	Present	Present	A	Absent	Normal
		R	Absent	Present	Present	As	Absent	Normal
ANCI_02	Male	L	Absent	Present	Present	A	Absent	CN abnormal
		R	Absent	Present	Present	A	Absent	CN abnormal
ANCI_03	Male	L	Absent	Present	Present	A	Absent	Normal
		R	Absent	Present	Present	A	Absent	Normal
ANCI_04	Male	L	Absent	Absent	Present	A	Absent	Normal
		R	Absent	Absent	Present	A	Absent	Normal
ANCI_05	Female	L	Absent	Present	Present	A	Absent	Normal
		R	Absent	Present	Present	A	Absent	Normal
ANCI_07	Male	L	Absent	Present	Present	A	Absent	Normal
		R	Absent	Present	Present	A	Absent	Normal
ANCI_09	Female	L	Absent	Absent	Present	A	Absent	Normal
		R	Absent	Present	Present	C	Absent	Normal
ANCI_10	Female	L	Absent	Present	Present	As	Absent	Normal
		R	Absent	Present	Present	As	Absent	Normal

L, left; R, right; ABR, auditory brain stem responses; DPOAEs, distortion-product otoacoustic emissions; CM, cochlear microphonic; MRI, magnetic resonance imaging; CN, cochlear nerve.

**Table 2:** Subjects’ general information.

Subject	Sex	Ear implanted	Age at HL(mo)	Age at CI(mo)	Type of implant
ANCI_01	M	R	Congenital	29	90k(Advanced Bionics, USA)
ANCI_02	M	R	6	14	CI24R CA(Cochlear, Australia)
ANCI_03	M	L	18	36	CI24R ST(Cochlear, Australia)
ANCI_04	M	R	14	48	CI24R CA(Cochlear, Australia)
ANCI_05	F	L	16	19	CI24R CA(Cochlear, Australia)
ANCI_07	M	R	7	13	90k(Advanced Bionics, USA)
ANCI_09	F	R	Congenital	40	CS-10A(Nurotron, China)
ANCI_10	F	R	Congenital	43	CI24R CA(Cochlear, Australia)

M, male; F, female; R, right; L, left; HL, hearing loss; CI, cochlear implantation.

**Table 3:** Subjects’ preoperative hearing thresholds.

Subject	Ear	250 Hz	500 Hz	1000 Hz	2000 Hz	4000 Hz	8000 Hz	4FA
ANCI_01	L	100	115	115	NR	NR	NR	117.50
	R	100	100	110	120	NR	NR	112.50
ANCI_02	L	CNT	CNT	CNT	CNT	CNT	CNT	-
	R	CNT	CNT	CNT	CNT	CNT	CNT	-
ANCI_03	L	85	95	105	105	115	NR	105.00
	R	75	95	95	100	110	NR	100.00
ANCI_04	L	90	95	100	110	115	NR	105.00
	R	90	95	100	105	110	NR	102.50
	L*	40	40	35	50	80	NT	51.25
	R*	25	25	35	55	NR	NT	58.75
ANCI_05	L	NR	105	95	100	105	NR	101.25
	R	75	90	90	100	100	90	95.00
ANCI_07	L	CNT	CNT	CNT	CNT	CNT	CNT	-
	R	CNT	CNT	CNT	CNT	CNT	CNT	-
ANCI_09	L	100	115	115	110	NR	NR	115
	R	90	105	110	100	95	85	102.50
	L*	65	65	50	55	NR	NT	72.5
ANCI_10	L	95	100	85	65	70	80	80.00
	R	80	75	95	85	65	65	80.00

All hearing thresholds are presented as dB hearing level. NR, no response at the highest output level; CNT, could not test; NT, not tested; 4FA, four-frequency average unaided threshold at 0.5, 1.0, 2.0, and 4.0 kHz; L, left; R, right; L* and R*, aided thresholds through hearing aids in the left and right ear, respectively. When the average thresholds were calculated, NR was observed at 120 dB hearing level, while CNT and NT were not averaged.

### Effect evaluation methods

Because of the variability among the subjects in their cooperation with completing the hearing and speech test, hearing sensitivity and speech recognition performances of all eight children were individually evaluated preoperatively and postoperatively. Aided hearing thresholds were tested via behavioral audiometry in sound fields using warble tones. ECAP was tested intraoperatively and postoperatively to evaluate neural activity. An ECAP response including both N1 and P1 peaks was considered as a valid ECAP waveform. To quantify the presentation of ECAP, the electrode array was divided into three parts according to the corresponding location in the cochlea after implantation: basal (electrodes #1–7), middle (electrodes #8–15), and apical (electrodes #16+). The ECAP of the subject was considered normal only when a valid ECAP waveform was recorded from at least one electrode in each part.

The assessment of speech recognition varied depending on the subject’s age at the time of implantation, hearing–speech experience, and ability to cooperate with the tests, including the Mandarin Early Speech Perception Test (MESP) [39, 40], MAIS, and IT-MAIS [41]. Similar to the English Early Speech Perception test, the MESP test is a step-up close-set assessment tool for early speech perception abilities in children with lexical abilities. The standard MESP is used for children who have a glossary of more than six years [39], and a low-verbal version of this test is used for children with poor speech development who pass only the first category of the standard version. The standard version is a norm-referenced and software-administered speech perception test that relies on recorded speech materials. It includes six category levels, each of which measures a different category of early speech perception. Categories 1, 2, and 3, measure speech sound detection, speech pattern perception, and spondee recognition respectively, while categories 4, 5, and 6 measure mandarin vowel perception, consonant perception, and tone perception, respectively. The low-verbal version MESP includes a training course and three levels of pattern, spondee, and monosyllable perception. The more difficult Mandarin Pediatric Speech Intelligibility (MPSI) [40] test of sentence recognition in quiet and noisy conditions was administrated if the speech perception abilities of the children were sufficient. The MAIS or IT-MAIS questionnaire concerning the child’s hearing skills was administered to the parents using software developed by the West China Hospital of Sichuan University and House Ear Institute (MAPP 3.14) [41]. The MAIS/IT-MAIS evaluates ten items in three dimensions: bonding to the device (two items), detection (i.e., alerting to sound, assessed by four items), and perception (i.e., deriving meaning from sound, assessed by four items). Each item was scored from 0 to 4, and final MAIS/IT-MAIS scores in each dimension were calculated by the software and referenced to birth-age norms. As obtaining an age-related norm for bonding to the device is difficult, the items in this dimension were not analyzed independently, but rather integrated into a total score with all the items. The recognition scores for Mandarin monosyllables, disyllables, and sentences in quiet and noisy conditions were tested in subjects capable of cooperating with regular speech audiometry.

Communication ability was reflected by speech production, which was evaluated by the Speech Intelligibility Rating (SIR). SIR scales the speech intelligibility of children into five categories by the parents under the instruction of an audiologist. The lowest level of SIR describes unintelligible connected speech, involving the use of pre-recognizable words, where the primary mode of communication may be manual. The highest level of SIR describes connected speech that is intelligible to all listeners, where the child is easily understood in everyday context [42, 43].

### Hierarchic assessment

The efficacy of cochlear implantation was evaluated using a stepwise hierarchic assessment for achieving: (1) effective audibility, such that thresholds were significantly improved and/or electrically evoked responses (including ECAP and EABR) were present; (2) improved speech recognition, which means better postoperative speech recognition scores than preoperative ones assessed via either questionnaires (including MAIS/IT-MAIS) or speech audiometry (including mandarin monosyllables, disyllables, and MESP); (3) effective speech, with improved sentence recognition and/or speech recognition ability in noisy conditions (via MPSI and Mandarin Hearing In Noise Test [44], among others); and (4) effective communication, with development of speech production, study capabilities, and social communication skills (via SIR and Meaningful Use of Speech Scale [45], among others). Results of different evaluation methods were integrated into this hierarchic evaluation frame.

### Ethics statement

The ethics committee of PLA General Hospital approved all procedures, and the study protocol conformed to the Declaration of Helsinki. All parents of the subjects provided written informed consent before data collection.

### Statistical analysis

Because of wide variation in the abilities of AN children to complete the hearing and speech tests, the analyses in the present study utilized a within-subjects and repeated-measures design. A Student’s *t* test was performed when the speech performance was evaluated via MAIS/IT-MAIS, and the significance was set at *P* < 0.05.

## Results

### General results

**Fig 1:**
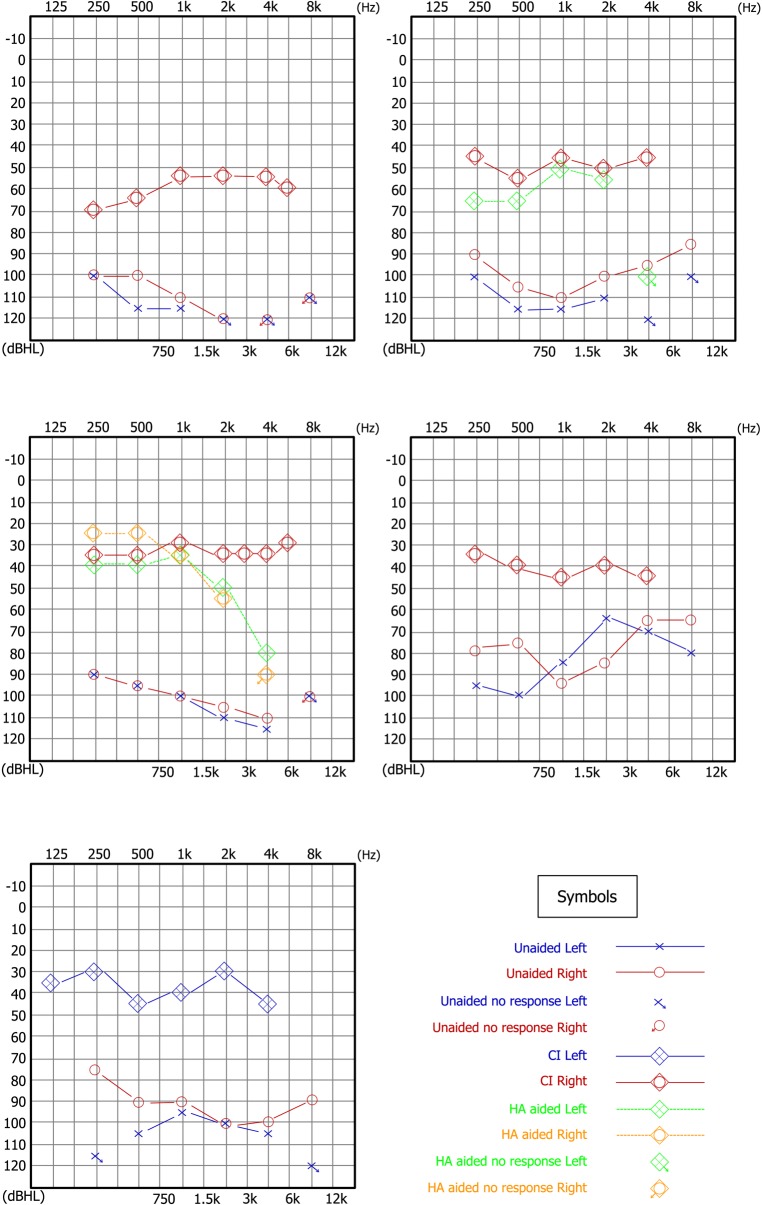
Preoperative and postoperative audiograms. (**a**) ANCI_01, preoperative and 10 months postoperative; (**b**) ANCI_04, preoperative and 7 months postoperative; (**c**) ANCI_05, preoperative and 48 months postoperative; (**d**) ANCI_09, preoperative and 11 months postoperative; (**e**) ANCI_10 preoperative and 12 months postoperative.

All subjects underwent successful implantation. The devices were switched on 7–30 d after implantation. Five subjects exhibited postoperative electrophysiologic responses, including ECAP (ANCI_01, 02, 03, 04, and 07) and EABR (Subject ANCI_02). Five children (ANCI_01, 04, 05, 09, and 10) who were able to cooperate for behavioral audiometry showed improvement in the thresholds of speech frequency by 30 to 70 dB, with an average postoperative threshold of 43.8 ± 10.2 dB HL. Both children who had used hearing aids demonstrated a 15- to 25-dB advantage with the CI over the hearing aids, particularly at frequencies > 2 kHz (Fig 1).

**Fig 2:**
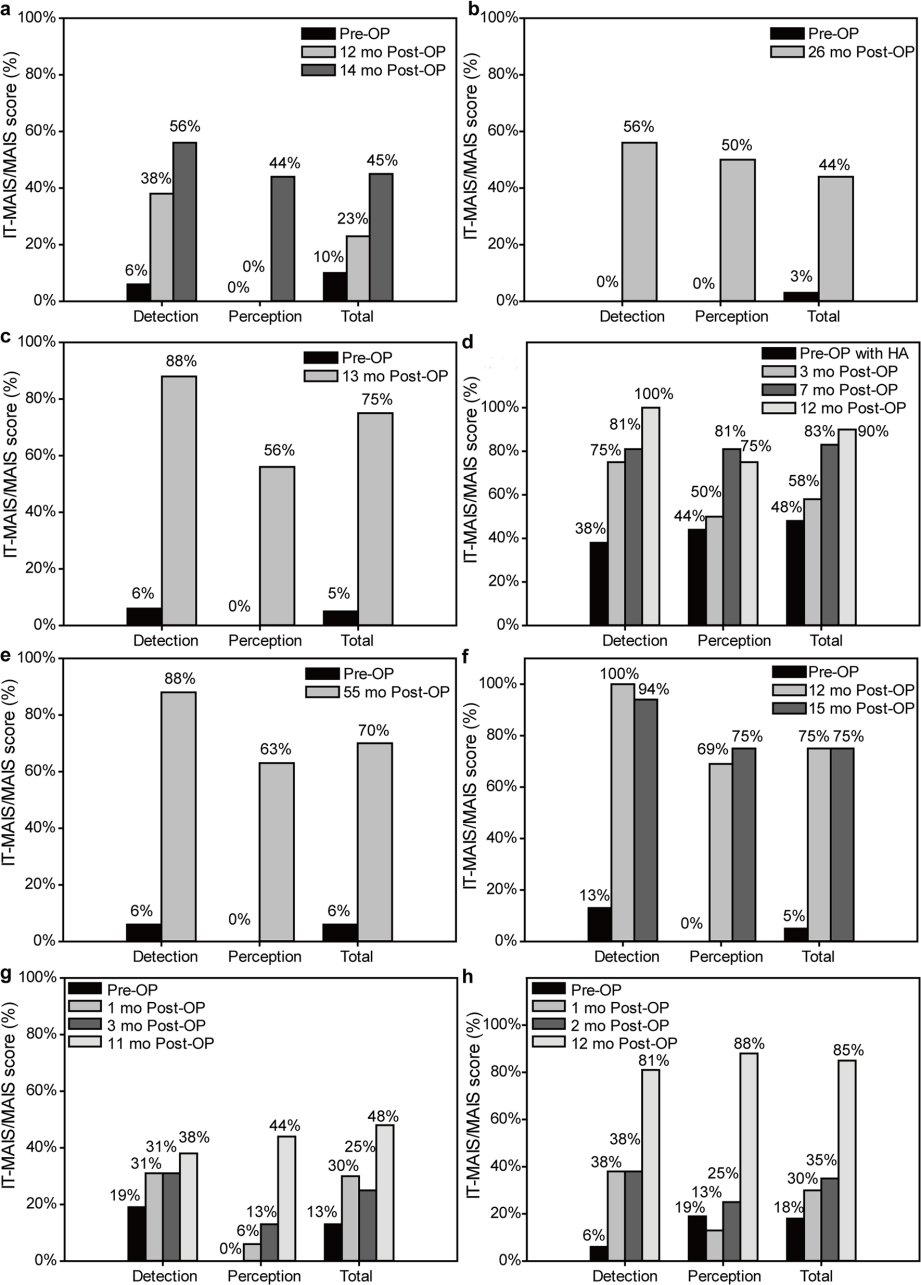
Progress according to IT-MAIS/MAIS scores. (**a**) ANCI_01, IT-MAIS/MAIS score; (**b**) ANCI_02, IT-MAIS/MAIS score; (**c**) ANCI_03, MAIS score; (**d**) ANCI_04, MAIS score; (**e**) ANCI_05, IT-MAIS/MAIS score; (**f**) ANCI_07, IT-MAIS/MAIS score; (**g**) ANCI_09, MAIS score; (**h**) ANCI_10, MAIS score. MAIS, Meaningful Auditory Integration Scale; IT-MAIS, Infant-Toddler Meaningful Auditory Integration Scale.

**Fig 3:**
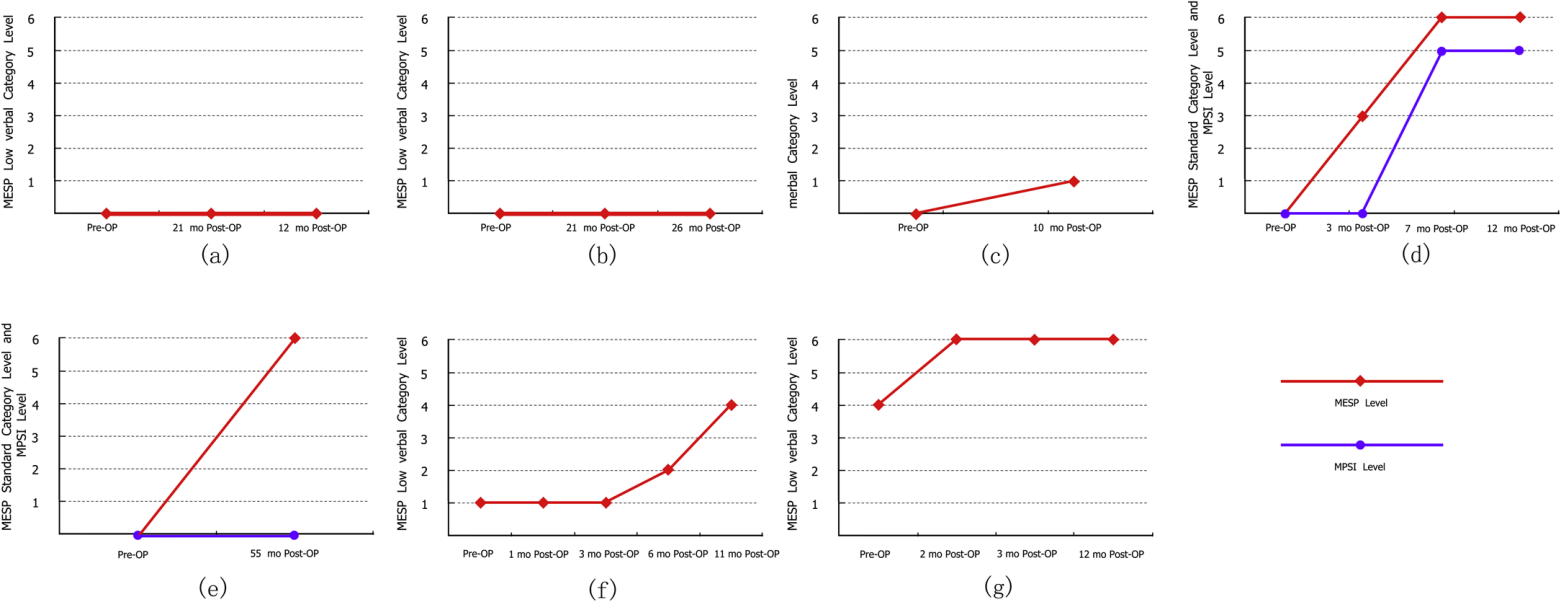
Progress according to MESP performance. (**a**) ANCI_01, MESP performance; (**b**) ANCI_02, MESP performance; (**c**) ANCI_03, MESP performance; (**d**) ANCI_04, MESP and Mandarin Pediatric Speech Intelligibility Test performance; (**e**) ANCI_05, MESP performance; (**f**) ANCI_09, MESP performance; (**g**) ANCI_10, MESP performance. MESP, Mandarin Early Speech Perception Test.

Speech performance varied drastically among the children. The average preoperative MAIS/IT-MAIS total, detection, and perception scores were 12.8 ± 14.7%, 11.8 ± 12.0%, and 7.9 ± 16.0%, respectively (Fig 2). Among these children, six underwent a one-year postoperative MAIS/IT-MAIS, with total, detection, and perception scores of 66.0 ± 25.6%, 73.2 ± 28.0%, and 56.3 ± 31.7%, respectively, which were significantly higher than the preoperative scores (all *P* < 0.01). Four children underwent a MAIS/IT-MAIS after more than one year. Overall postoperative total, detection, and perception scores of MAIS/IT-MAIS for all eight children on the final test session were 66.5 ± 18.4%, 75.1 ± 22.2%, and 61.9 ± 14.7%, respectively, which were significantly higher than the preoperative scores (all *P* < 0.0001). Seven of the eight children cooperated during performance of the MESP. Two children (ANCI_04 and 05) passed all categories in the standard version. Three children (ANCI_03, 09, and 10) made progress in the low-verbal version, but could not complete the standard version. The remaining two children (ANCI_01 and 02) did not progress past the first category (“cannot test”) in the low-verbal version (Fig 3).

### Hierarchic results

Table 4 shows the hierarchic results of the last evaluation session. All eight prelingual children (100%) obtained effective audibility with substantially improved hearing thresholds or normal ECAP/EABR. Five subjects (62.5%) reached the level of improved speech with improved MAIS/IT-MAIS scores and MESP performance. One subject (12.5%) reached the level of effective speech, and was the only subject with marked progression in sentence recognition under the noisy condition as determined by the MPSI test. None (0%) of the prelingual children performed at the level of effective communication; all of their SIR scores stayed at the preoperative lowest level 1, indicating that none of these children developed effective speech-producing ability or further social communication skills.

**Table 4:** Stepwise hierarchic assessment of efficacy of cochlear implantation.

Subject	Effective audibility	Improved speech recognition	Effective Speech	Effective communication	Last postoperative evaluation(mo)
ANCI_01	★	○	○	○	14
ANCI_02	★	○	○	○	26
ANCI_03	★	★	○	○	13
ANCI_04	★	★	★	○	12
ANCI_05	★	★	○	○	55
ANCI_07	★	○	○	○	15
ANCI_09	★	★	○	○	11
ANCI_10	★	★	○	○	12

★, subject in this row has approached the level of the current column; ○, subjects in this row is not approaching the level of the current column.

Subject ANCI_04 performed the best among all eight children. In contrast, Subjects ANCI_01 and ANCI_02 remained at the level of effective audibility by the time of data pooling. These differences in hierarchic results reflect the different benefits provided by the CI to the various AN individuals.

## Discussion

### Performance in AN children after cochlear implantation

The general results of this study demonstrate that CIs provide definite improvement in audibility, as evidenced by either behavioral audiometry or electrophysiologic results. Children who were capable of completing behavioral audiometry showed significant improvement in hearing thresholds. Similar to data reported by Roush *et al*. [11], the average thresholds among prelingual recipients with AN in our cohort were 40–50 dB better than the preoperative thresholds, and 15–25 dB better than that achieved with hearing aids, particularly at frequencies > 2 kHz. Such results demonstrate that, although CIs are associated with more risk factors than hearing aids (e.g., general anesthesia, surgical wound, and risk of infection), they can provide greater benefit to AN children than hearing aids. However, audibility is not the primary impairment in subjects with AN.

In this study, there was large variability in postoperative speech recognition performance among prelingual recipients with AN, as has been reported in individuals with SNHL [46]. Some subjects did not develop open-set recognition after long-term use of the CI, despite having normal behavioral pure-tone thresholds (e.g., ANCI_01) or presenting electrically neural responses (e.g., ANCI_02 and 07). In prelingual AN recipients, preoperative and postoperative hearing training plays as important a role as it does in SNHL individuals. The one-year MAIS/IT-MAIS scores and MESP performance of subject ANCI_04, who underwent hearing–speech training both before and after implantation, were substantially better than subject ANCI_03, who underwent no training. Subjects ANCI_05 and 07 made no progress in hearing–speech performance until after joining rehabilitation training following implantation. Collectively, these results highlight the importance of pre- and postoperative hearing experience, which might affect outcomes of AN recipients.

CIs are widely used to improve audibility and speech communication in individuals with profound to severe SNHL, though variability in outcomes has also been reported, particularly in pediatric recipients [46, 47]. However, the variability observed in AN recipients may be related not only to the hearing–speech experience, but also to the uncertainty regarding the site of lesion, which might result in unpredictable performance after implantation [14, 15, 17–19, 28–30, 32, 33]. Considering this unpredictability, only a small proportion of AN individuals in China undergo cochlear implantation [48], which also likely represents a financial burden for the family. In addition, there is wide variation in the abilities of AN children to sufficiently cooperate in order to complete the hearing and speech tests, as observed in the present study. Thus, data from AN subjects cannot be pooled, as it is for studies involving SNHL subjects. Because of this, the analyses in the present study utilized a within-subjects and repeated-measures design. Indeed, this complicates the quantification of benefit gained by cochlear implantation in AN children. For this reason, we hypothesized that a stepwise hierarchic assessment, which integrated a diversity of assessment tools, would provide a better evaluation of CI efficacy in AN children. In the current study, MESP, MAIS, and IT-MAIS were used concurrently, as were open-set spondee, monosyllable, and sentence recognition tests in both quiet and noisy conditions in individuals with good performance, such as subject ANCI_04.

### Determining benefits of CIs in AN individuals using hierarchic assessment

Various methods and criteria have been used to evaluate the effect of CIs in AN individuals, such as neural responses evoked via CI [15, 18, 19, 34, 35], improved hearing thresholds [19, 33], and improvements in speech recognition performance [16, 21–23, 25, 36–38]. The hierarchic assessment used in the present study was designed to be an open frame that can integrate different subjective or objective methods. This method is similar to those of the Functional Auditory Performance Indicators [49] and Functional Communication Measures [50]. The Functional Auditory Performance Indicators can generate a functional profile of a child’s auditory skills after administering all seven hierarchic categories. The Functional Communication Measures, on the other hand, include a series of seven-point rating scales that were developed by the American Speech-Language-Hearing Association to describe the different aspects of an adult’s functional communication and swallowing abilities over the course of an intervention. It is important to note that the hierarchic assessment used in the present study is not an independent evaluation procedure, but rather an integration and classification of existing performances and test results that allows for a convenient clinical evaluation of the effects of cochlear implantation. Instead of determining whether the CI is effective or ineffective for subjects with AN, this hierarchic assessment provides a more detailed classification of the subject’s hearing and speech performance.

The four hierarchies of the present assessment method reflect the step-up levels of hearing performance. The purpose of CIs is to obtain effective audibility (level one), which can be demonstrated through electrophysiologic tests or pure tone audiometry. Speech efficacy is the second level, and is difficult to assess. Two sublevels of speech performance were therefore established, a lower level representing an improvement over the preoperative performance regardless of the absolute speech recognition ability, and a higher level that requires either an absolute speech recognition ability that corresponds with that of an age-matched SNHL group or meaningful open-set scores for speech in the noisy condition. These two sublevels provide detailed descriptions and classifications of the speech performance of subjects, which is convenient for analysis of the irregular and within-subjects data from AN recipients. The highest objective level of hearing intervention is the achievement of effective social communication, upon which few studies in AN individuals have focused [11]. Importantly, the herein-described hierarchic assessment is not specific for AN individuals, and can be applied for assessing CIs in other groups, including SNHL subjects, as well as small-sized subject groups with irregular data.

Although the sample size of the present study was small, the data provide clinical evidence that may serve as a basis for future meta-analyses. A longer period of time may be required to demonstrate the efficacy of CIs in different individuals with AN. Therefore, a long-term follow-up period is needed to demonstrate the dynamic efficacy of CI in prelingual children with AN.

## Conclusions

Cochlear implantation benefits prelingual children with AN to different extents. The audibility of prelingual children with AN can be reconstructed with implantation, whereas the postoperative speech recognition performance varies among individuals. In the present study, some individuals demonstrated improved speech recognition and others achieved effective speech recognition in the noisy condition. Other subjects did not make progress in speech recognition, and no subject demonstrated effective social communication. Hierarchic assessment can provide a more proper framework by which to determine a subject’s benefits from the CI via a within-subjects and repeated-measures design.
